# Dosimetric analysis of six whole-breast irradiation techniques in supine and prone positions

**DOI:** 10.1038/s41598-024-65461-y

**Published:** 2024-06-21

**Authors:** Dong Wook Kim, Chae-Seon Hong, Junyoung Son, Se Young Kim, Ye-In Park, Mijoo Chung, Weon Kuu Chung, Min Cheol Han, Jihun Kim, Hojin Kim, Jin Sung Kim

**Affiliations:** 1 Department of Radiation Oncology, Yonsei Cancer Center, Heavy Ion Therapy Research Institute, Yonsei University College of Medicine, 50-1, Yonsei-Ro, Seodaemun-Gu, Seoul, South Korea 03722; 2 Department of Radiation Oncology, Yongin Severance Hospital, Yongin, South Korea; 3 Department of Radiation Oncology, Yonsei Cancer Center, Seoul, South Korea; 4 Department of Radiation Oncology, Changwon Hanmaeum Hospital, Hanyang University College of Medicine, Changwon, South Korea; 5 Department of Radiation Oncology, Kyung Hee University Hospital at Gangdong, Seoul, South Korea; 6 Department of Radiation Oncology, Gangnam Severance Hospital, Yonsei University College of Medicine, Seoul, South Korea

**Keywords:** Radiotherapy, Breast cancer, Quality of life, Radiotherapy

## Abstract

In breast cancer radiation therapy, minimizing radiation-related risks and toxicity is vital for improving life expectancy. Tailoring radiotherapy techniques and treatment positions can reduce radiation doses to normal organs and mitigate treatment-related toxicity. This study entailed a dosimetric comparison of six different external beam whole-breast irradiation techniques in both supine and prone positions. We selected fourteen breast cancer patients, generating six treatment plans in both positions per patient. We assessed target coverage and organs at risk (OAR) doses to evaluate the impact of treatment techniques and positions. Excess absolute risk was calculated to estimate potential secondary cancer risk in the contralateral breast, ipsilateral lung, and contralateral lung. Additionally, we analyzed the distance between the target volume and OARs (heart and ipsilateral lung) while considering the treatment position. The results indicate that prone positioning lowers lung exposure in X-ray radiotherapy. However, particle beam therapies (PBTs) significantly reduce the dose to the heart and ipsilateral lung regardless of the patient’s position. Notably, negligible differences were observed between arc-delivery and static-delivery PBTs in terms of target conformity and OAR sparing. This study provides critical dosimetric evidence to facilitate informed decision-making regarding treatment techniques and positions.

## Introduction

Breast cancer is the most frequently diagnosed malignancy and a leading cause of mortality among women^1,2^. Modern multidisciplinary treatment strategies, encompassing surgical resection, postoperative radiation, and preoperative or postoperative systemic therapy, have significantly enhanced the long-term survival prospects of breast cancer patients^3^. Notably, radiation therapy following breast-conserving surgery has exhibited outcomes on par with mastectomy^4,5^, especially for early-stage cases; this has led to an increased emphasis on improving patients’ long-term quality of life while minimizing radiation-induced malignancy.

The primary side effects of concern in breast cancer radiotherapy patients are radiogenic heart and lung damage^6–11^. A study by Darby et al. reported a linear increase of 7.4% per Gray (Gy) in the rate of major coronary events concerning the mean heart dose^7^. Furthermore, the reduction of the dose on the left anterior descending (LAD) artery has been shown to decrease the risk of radiation-induced stenosis in the heart^12^. Research has revealed that specific regions of the heart, including the heart apex or portions of the LAD artery, may receive high doses (up to 47.2 Gy) despite a low mean heart dose (below 3 Gy)^13^. Additionally, the risk of secondary cancer, often arising from irradiation of the contralateral breast or lungs, is a critical consideration^14,15^. For patients who survive beyond five years, breast cancer radiation therapy has been associated with a 14% increase in contralateral breast cancer risk, with younger patients and longer latency periods posing a higher risk^15–17^.

The clinical application of breast cancer radiation therapy has evolved from early three-dimensional conformal radiation therapy (3DCRT) to more precise techniques such as intensity-modulated radiotherapy and volumetric-modulated arc therapy (VMAT)^18–20^. Proton and carbon-ion radiation therapy (CIRT) has emerged as techniques that minimize dose deposition outside the clinical target volume (CTV)^21,22^. Additionally, the research on arc-delivery particle beam therapy (arc-delivery PBT), which holds promise for providing enhanced robustness, has been documented^23,24^. Studies have examined dosimetric variations among different treatment techniques to mitigate long-term radiation side effects in breast cancer radiation therapy and have assessed the effects of CTV size, organs at risk (OARs), target distances, treatment positions, and other factors on treatment planning^25–32^. However, there has been limited focus on arc-delivery PBT, including CIRT.

The current study aims to identify the optimal modality by comparing the dose characteristics of six external beam whole-breast irradiation techniques: 3DCRT, VMAT, intensity-modulated proton therapy (IMPT), proton arc therapy (PAT), intensity-modulated carbon-ion therapy (IMCT), and carbon arc therapy (CAT). We investigate the impact of treatment position, breast size, and the heart’s proximity to the breast on dose characteristics and the risks associated with secondary cancer.

## Materials and methods

### Patient selection and computed tomography (CT) simulation

We prospectively enrolled fourteen breast cancer patients, evenly divided between left-sided and right-sided cases, who had undergone adjuvant whole-breast irradiation with free breathing. This study was approved by the institutional review board of the Kyung Hee University Hospital at Gangdong (KHNMC 2017-02-004), and all procedures were performed in accordance with the relevant guidelines and regulations. Written informed consent was obtained from all patients. While the standard patient position for breast cancer CT scans at the hospital in the present study was supine, we performed non-contrast CT scans in both supine and prone positions with the arms raised to gather additional information for effective radiotherapy planning. In the prone position, the contralateral breast was gently moved aside, away from the ipsilateral breast. The CT simulator (Brilliance 64, Philips Medical Systems, The Netherlands) utilized for planning CT scans featured a 3-mm slice thickness, and the resulting image set was imported into the RayStation (RaySearch Laboratories AB, Stockholm, Sweden) treatment planning system (TPS).

### Planning parameters

The planning target volume (PTV) was defined as the breast contours with a 5-mm margin subtracted from the skin. Ipsilateral and contralateral lungs, the heart, and the contralateral breast were outlined as OARs. The prescribed dose was 50.4 Gy delivered in 28 fractions, covering 95% of the PTV. In the supine and prone positions, the PTV sizes ranged between 161–890 cc and 172–1156 cc, respectively. All plans were optimized to ensure target coverage and adhere to OAR dose constraints. For PTV, we required that 100% of the prescription doses cover over 95% of the PTVs, with the maximum dose limited to less than 110%. Regarding the ipsilateral lung, the V20Gy and V10Gy were kept below 10% and 20%, respectively, while the maximum point dose to the spinal cord was capped at 45 Gy. The maximum point dose to the heart was constrained to less than 7.5 Gy, and to mitigate the risk of secondary cancer, the volume of the heart receiving 1.0 Gy was limited to below 0.5%. Similarly, to reduce secondary cancer risks, the maximum point dose to the contralateral breast was restricted to less than 0.5 Gy. The maximum point dose was capped at less than 51.5 Gy for the ipsilateral chest wall, and for the contralateral chest wall, it was limited to less than 1.0 Gy. For robust optimization in particle beam therapy planning, the isotropic universal position uncertainty and range uncertainty were set at 5 mm and 3.5%, respectively, within RayStation TPS^33,34^. In CIRT planning, the relative radiobiological effectiveness (RBE) dose was computed using the modified microdosimetric kinetic model^35–37^.

**Figure 1:**
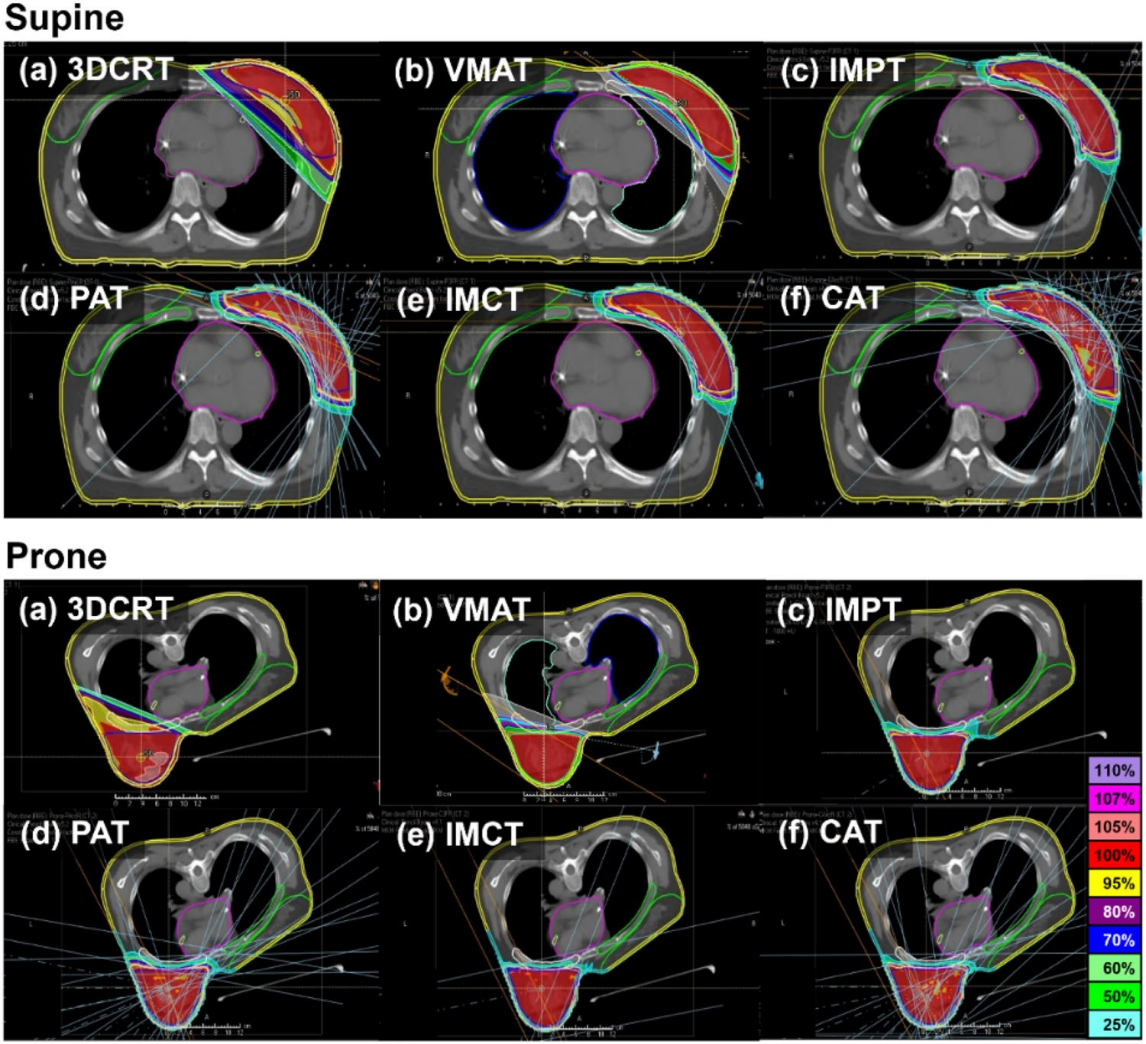
Axial dose distribution in supine (top) and prone positions (bottom) for the same patient: (**a**) 3DCRT, (**b**) VMAT, (**c**) IMPT, (**d**) PAT, (**e**) IMCT, and (**f**) CAT.

The 3DCRT plans utilized two tangential fields, while the VMAT plans comprised two arcs. Each collimator angle was chosen to provide additional degrees of freedom, enhancing the plan's quality. X-ray radiotherapy (XRT) treatment was implemented using the 6 MV photon beam from Electa VERSA-HD (Electa AB, Stockholm, Sweden) linear accelerator equipped with an Agility 160 multileaf collimator. Static-field particle beam therapy (static-field PBT) plans involved three field beams. In the supine position, the gantry angles for static-field PBT were 330°, 30°, and 90° for the left breast and 270°, 330°, and 30° for the right breast. In the prone position, these angles were 150°, 190°, and 240° for the left breast and 110°, 180°, and 220° for the right breast. The gantry angles for arc-delivery PBT ranged between 320°–110° and 50°–270° for the left breast and between 250°–40° and 100°–220° for the right breast in the supine and prone positions, respectively. As RayStation TPS (v10A) does not support arc-delivery PBT planning, we conducted simulations by arranging 13 to 16 beams consecutively at 10° intervals. The spot spacing ranged from 0.5 to 1.5 cm for the proton beam and 0.25 to 0.5 cm for the carbon-ion beam. Figure 1 illustrates the dose distributions for the six different external beam whole-breast irradiation plans in both supine and prone positions.

### Comparison of dose characteristics based on dose-volume histograms (DVHs)

We employed DVHs, isodose distributions, and dosimetric indices for the lung, heart, LAD, contralateral breast, and skin to assess the quality of the treatment plans. The radiotherapy plans were evaluated with respect to the Homogeneity Index (HI) and Paddick Conformity Index (PCI) of the PTV^38^. HI provides insight into dose uniformity and is defined as HI = (|D2 − D98|/Rx) × 100, where D2 and D98 represent the doses received at 2% and 98% of the PTV, respectively, and Rx is the prescription dose^39^. Conversely, PCI describes dose conformity and is defined as PCI = (V95PTV × V95PTV)/(VPTV × V95), where V95PTV and V95 denote the volumes receiving a dose greater than 95% of the Rx within the PTV and the entire body, respectively^40^. Furthermore, we compared the mean lung dose and V20Gy to assess the risk of pneumonitis. We compared the mean heart dose and the maximum LAD dose to evaluate the probability of radiation-induced side effects, such as cardiovascular risk. Furthermore, we estimated and compared the mean contralateral breast dose and D2 at the skin.

### Distance between target and OARs (lung and heart) considering treatment position

We examined the geometric variances in the supine and prone positions concerning the target and OARs. In particular, we assessed changes in the PTV volume and the minimum distance (the shortest distance from the PTV surface to the ipsilateral lung or heart) between the target and OARs based on the treatment position.

### Secondary cancer risk estimates

The report on the Biological Effects of Ionizing Radiation (BEIR VII)^41,42^ with Schneider’s OED concept, which included the impact of fractionation and incorporated a repair and population parameter quantified excess absolute risk (EAR) for an individual exposed to radiation at a dose (D) at age (e) using the following formula:

1 $$\text{EAR }=OED{\beta }{\prime}\text{exp}\left[{\gamma }_{e}\left(agex-30\right)+{\gamma }_{a}\text{ln}\left(\frac{agea}{70}\right)\right]$$

where OED is the organ equivalent dose, β` represents the initial slope of the dose–response curve relationship of secondary cancer induction, *agea* is the attained age from the exposed age *agex*, and γ_e_ and γ_a_ denote age-modifying factors. All parameters utilized for calculating Excess Absolute Risks (EARs) were sourced from Schneider's work^42^. The initial computation of EARs was conducted for patients based on their actual ages of irradiation, under the presumption that they would live to be 70 years old. To mitigate the influence of varying irradiation ages on EAR findings, a standardization process was implemented by recalculating EARs for a specific cohort. This cohort consisted of individuals irradiated at the age of 30, with projections indicating they would achieve an age of 70. This adjustment allows for a more consistent comparison of EARs across different patient groups.

Organ equivalent dose (OED) was used to calculate EAR, utilizing a plateau dose–response model for estimating the OED of the contralateral breast as follows:

2 $${OED}_{plateau}= \frac{1}{V}\sum_{i}{V}_{i}(\frac{1-\text{exp}(-\delta {D}_{i})}{\updelta })$$

Upon the refinement of the plateau model, coupled with the consideration of the number of fractions in fractionated therapy, the OED for an organ, as per the full mechanistic model, was determined as follows:

3 $${OED}_{mechanistic}= \frac{1}{V}\sum_{i}{V}_{i}\left(\frac{1-\text{exp}\left(-\delta {D}_{i}\right)}{\delta \text{R}}\right)[ 1-2R+ {R}^{2}\text{exp}\left(-\delta {D}_{i}\right)- {\left(1-R\right)}^{2}\text{exp}(-{\frac{\delta \text{R}}{1-R}D}_{i}) ]$$

where *V* represents the total volume of the organ at risk (including contralateral breast, ipsilateral lung, and contralateral lung) and *Vi* denotes a specific volume element that is subjected to an exposed dose element *Di*^42,43^. For the full mechanistic model, the parameter δ was determined to be 0.044 Gy^−1^ with R equaling 0.15 for the female breast, and δ is 0.042 Gy^−1^ with R set at 0.83 for the lung. These parameters were derived from a combined fit to the data from atomic bomb survivors and Hodgkin’s disease patients treated with single doses ranging from 2 to 40 Gy assuming an alpha/beta (α/β) value of 3 Gy.

### Statistical analysis

To compare the dosimetric parameters and geometric distances for these treatment plans, we conducted paired two-tailed t-tests, with Bonferroni correction applied (utilizing SPSS, v26, Chicago, USA). The data were presented as mean values with standard deviations, and statistical significance was considered at *p* < 0.05.

## Results

A total of 168 treatment plans from 14 patients were evaluated in this study. Figure 1 illustrates an example of axial CT images with dose distributions in the supine and prone positions of a patient for six treatment techniques. The dosimetric results of PTV and OARs for the treatment techniques are presented in Supplementary Table S1.

### Dosimetric comparison of treatment techniques

**Figure 2:**
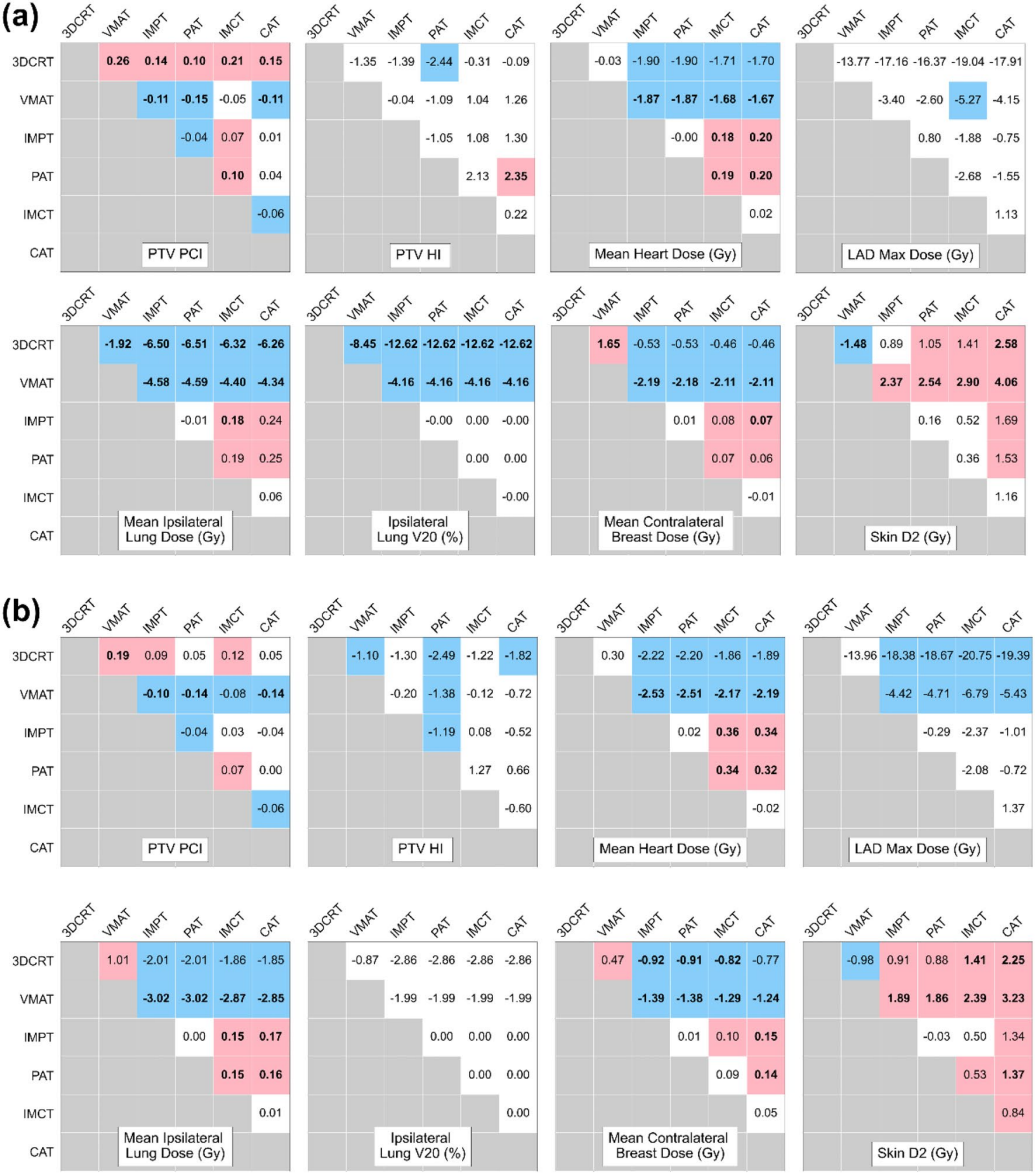
Differences in dosimetric parameter values in (**a**) supine and (**b**) prone positions. Pink cells indicate significant positive differences, blue cells indicate significant negative differences (p < 0.05), and strongly statistically significant (p < 0.001) values are presented in bold.

Figure 2 displays the mean target coverage and differences in OAR doses among 14 patients for the six techniques. VMAT exhibited superior PCI coverage, albeit it recorded the highest mean dose to the contralateral breast. Notably, statistically significant reductions in mean heart dose, mean lung dose, and mean contralateral breast dose were observed with PBT compared to XRT (*p* < 0.05). However, a proclivity toward higher skin dose was noted in the case of PBT. Among the PBT techniques, proton therapy, encompassing IMPT and PAT, offered a lower mean heart dose compared to CIRT, which comprises IMCT and conventional active scanning CAT. Nevertheless, it is crucial to highlight that the average mean heart dose for all techniques remained below 0.5 Gy (RBE). Arc-delivery PBT yielded no benefits in terms of OARs sparing for the heart, ipsilateral lung, and contralateral breast compared to static-field PBT, and the target conformity was inferior (p < 0.05).

### Dosimetric comparisons between supine and prone positions

**Figure 3:**
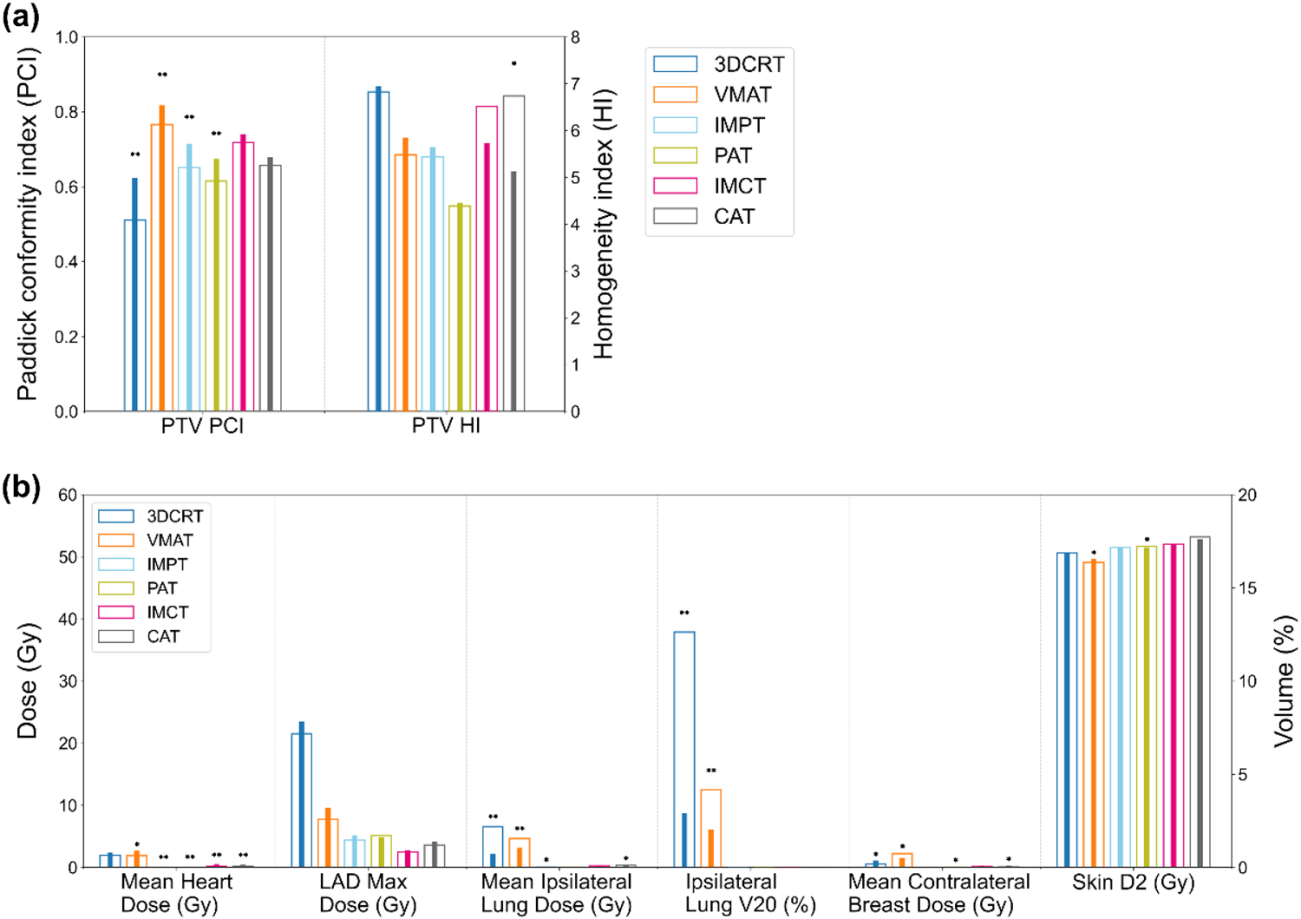
Differences in average dosimetric parameters between the supine (wide bar) and prone (narrow bar) positions for PTV (**a**) and OARs (**b**). Asterisks indicate statistically significant differences: *p < 0.05; **p < 0.001.

Figure 3 illustrates the mean differences in dosimetric parameters for PTV and OARs between the supine and prone positions. In the prone position, the PTV's PCI surpassed that in the supine position for all treatment techniques. However, statistical significance was only observed in the case of XRT and proton therapy. Conversely, the supine position resulted in a statistically significant reduction in the mean heart dose for most treatment techniques. Furthermore, in the context of XRT, the prone position led to decreased mean lung dose and V20 values for the ipsilateral lung. In contrast, for PBT, these values remained minimal, regardless of the patient's position.

### Distance between target and OARs (lung and heart) considering treatment position

**Figure 4:**
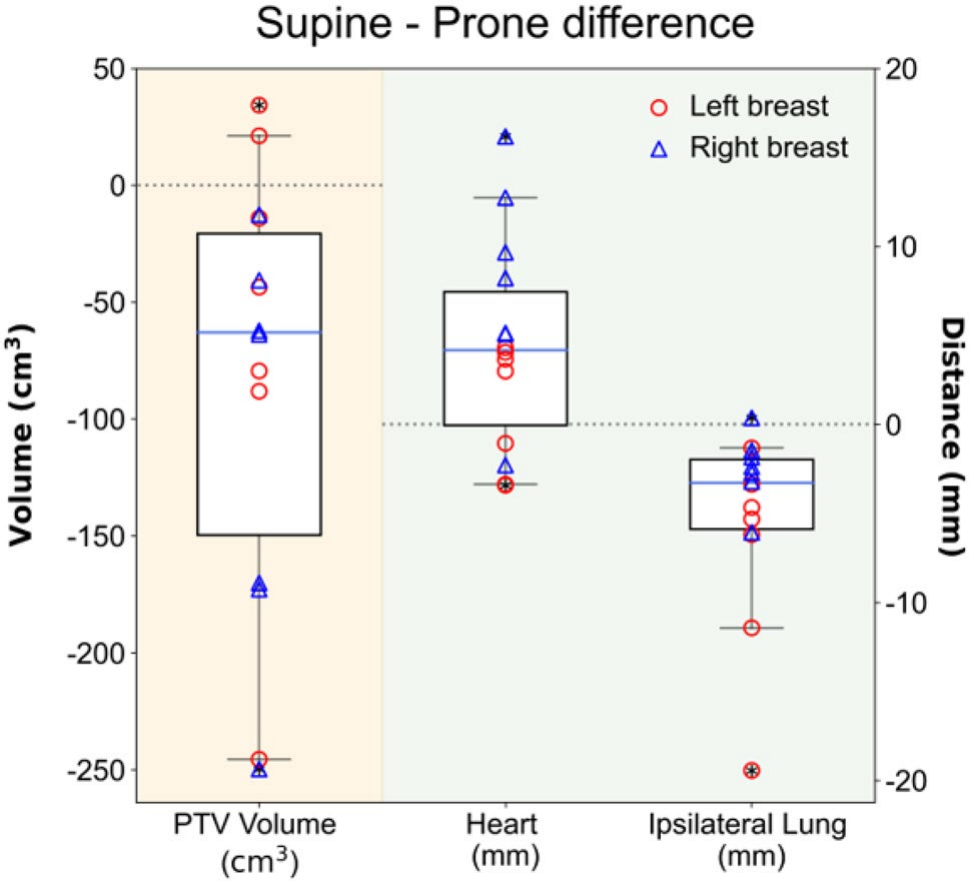
Differences in the PTV volume and separation distance between the supine and prone positions. Separation distance was defined as the shortest three-dimensional distance from the 3D contours of PTV and OARs. Red circle and blue triangle marks represent the left- and right-sided breast cancer patients, respectively. Light orange and light green shaded areas correspond to PTV volume and separation distance of OARs from PTV, respectively.

The difference in geometric distances between the PTV and OARs, specifically the lung and heart, depending on the patient's treatment position, are depicted in Fig. 4 and summarized in Supplementary Table S2. In terms of the separation between the PTV and OARs, it was observed that the supine position led to a more substantial separation effect for the heart, while the prone position allowed the ipsilateral lung to separate more from the PTV as compared to the supine position (*p* < 0.05).

### Estimation of secondary cancer risk

**Figure 5:**
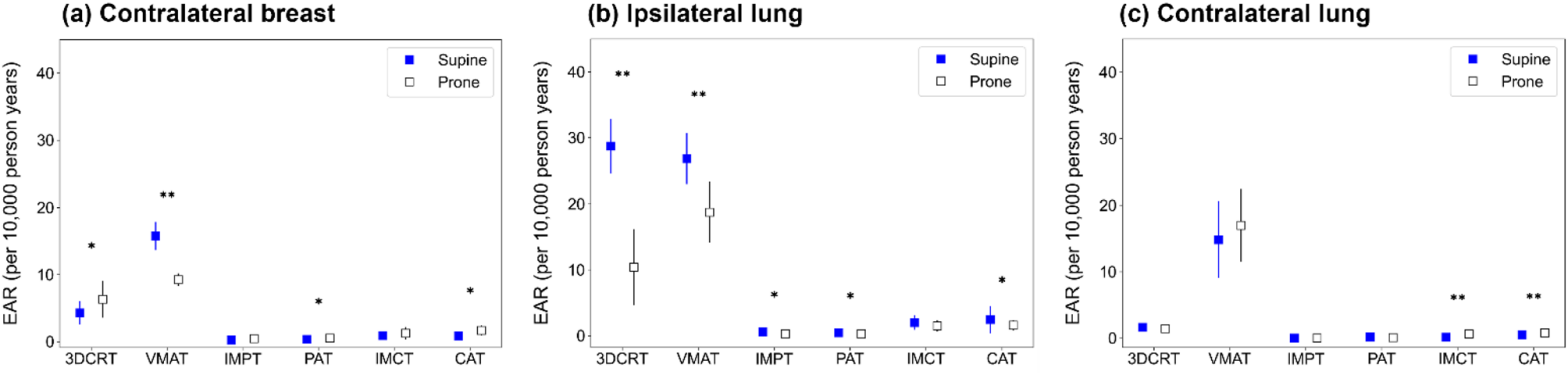
Mean excess absolute risk (EAR) for the contralateral breast (**a**), ipsilateral lung (**b**), and contralateral lung (**c**) in supine and prone positions for 3DCRT, VMAT, IMPT, PAT, IMCT, and CAT. Asterisks indicate statistically significant differences: *p < 0.05; **p < 0.001.

Figure 5 illustrates the differences in the mean EAR for the contralateral breast, ipsilateral lung, and contralateral lung in the supine and prone positions. VMAT yielded the highest mean EAR estimate, followed by 3DCRT. Statistically, the mean EARs in PBT were significantly lower than those for XRT. No statistical significance was found between static-field PBT and arc-delivery PBT. Furthermore, in the supine position, proton therapy better reduced the mean EAR than CIRT (Supplementary Table S3).

## Discussion

Advanced treatment techniques such as PBT and the use of prone positions can effectively reduce the radiation doses delivered to adjacent OARs while maintaining the same level of disease control in breast cancer patients. To support clinical decision-making, we conducted a thorough analysis of the dosimetric evidence that underlies the potential advantages of radiotherapy. We also evaluated the dosimetric efficacy of these techniques based on treatment positions.

PBT significantly lowered the mean heart dose, mean lung dose, and mean contralateral breast dose compared to XRT. This outcome is attributed to the excellent depth-dose distributions of PBT, allowing for a substantial reduction in OAR doses while preserving target coverage. Prior research has consistently reported that proton therapy offers significant advantages in sparing the heart and lungs compared to XRT^25–28^. Ischemic heart disease can persist for up to 20 years after radiation exposure, and there is a proportional increase in coronary events with the mean dose to the heart, without a clear cutoff value. Therefore, to reduce the incidence of coronary events, minimizing the mean heart dose to the greatest extent possible is crucial^7^. Vega et al. suggested proton therapy as a cost-effective measure for patients with cardiac risk factors for mean heart dose ≥ 5 Gy^44^. A recent study has established a direct correlation between low-dose radiation (specifically, V5) and the occurrence of acute coronary events. Thus, the increase in heart volume exposed to radiation should be reduced^45^. Notably, our study revealed that the mean heart dose in PBT was consistently less than 0.5 Gy (RBE), indicating its potential to mitigate radiation-related cardiac morbidity. The use of CIRT breast cancer treatment has been primarily limited due to the scarcity of facilities offering this treatment and the associated high costs^46^. Moreover, the eligibility criteria for CIRT generally target low-risk stage I breast cancer cases, which are typically amenable to partial breast irradiation^47,48^. This study represents the first comparative dosimetric analysis of CIRT in the context of whole-breast irradiation. Interestingly, our results showed similarities between CIRT and proton therapy in terms of sparing OARs. Consequently, CIRT may present an attractive option for treating patients with a high locoregional recurrence rate or unresected nodal disease in specific areas such as the supraclavicular fossa or internal mammary nodes^21^. However, further clinical investigations are needed to fully understand the role of CIRT in various treatment settings, including whole-breast irradiation.

This study compared static and arc delivery in XRT and PBT to evaluate the dosimetric effects of beam delivery methods. In XRT, VMAT demonstrated superior target dose conformity when compared to 3DCRT. However, VMAT resulted in higher mean doses and elevated secondary cancer risk for the contralateral breast (as indicated in Figs. 3 and 5). In general, VMAT can reduce the dose to the heart and ipsilateral lung while delivering a more uniform dose distribution to the target compared to 3DCRT. However, VMAT produces higher mean and low-dose volumes in OARs^49–52^. Arc-delivery PBT offers several benefits, including reduced calculation complexity and uncertainties, as well as optimization of high linear energy transfer deposition in tissues^53^. Arc-delivery PBT can be more robust than static-field PBT as range uncertainty is spread between different beam angles, and the RBE is increased within the target^53–56^. This approach has gained attention primarily due to the utilization of rotating gantries and spot-scanning techniques^54^. In particular, spot-scanning proton arc (SPArc) can provide better target coverage and a significantly lower dose of OARs across various sites in comparison to IMPT^54,55^. Notably, a study by Chang et al. underscored that SPArc could achieve superior OAR sparing and ensure robust plan quality, particularly in the context of left-sided WBRT compared to IMPT^24^. However, the results of our study did not indicate any discernible benefits of arc-delivery PBT for both proton therapy and CIRT. This discrepancy can be attributed to several methodological distinctions between the two studies. First, our study employed three beam angles for IMPT and 13–16 beam angles for arc therapy, whereas the study by Chang et al. utilized one beam for IMPT and 39 beams for SPArc^24^. Increasing the number of beams can potentially improve the dose gradient^57^. Another difference is that arc-delivery PBT in our study was generated by combining multiple fixed-angle beam. Conversely, SPArc was based on an algorithm that optimized the number of arc control points and energy layers delivered from each angle^53^. Dynamic arc delivery, such as SPArc, where the gantry rotates dynamically during dose delivery, can improve dosimetric quality compared to multiple fixed-angle beam delivery. Dynamic arc therapy uses hundreds of beam angles with an energy layer selection algorithm to optimize the RBE inside the target volume, thereby increasing the biological effectiveness of particle therapy. This method offers additional degrees of freedom in optimization and delivery, potentially streamlining the treatment process with a single arc trajectory^24^. However, current limitations in hardware, beam control systems, and treatment planning systems restrict the clinical and practical application of dynamic particle arc therapy. Consequently, the multiple fixed-angle beam delivery used in this study serves as an alternative feasible technique but should not be equated with dynamic arc delivery. Therefore, caution is necessary when interpreting the results of this study using multiple fixed-angle particle beams in terms of dynamic arc delivery. Further research is required to compare multiple fixed-angle beam delivery with dynamic arc delivery planning and better understand the clinical significance and application of arc-delivery PBT.

**Figure 6:**
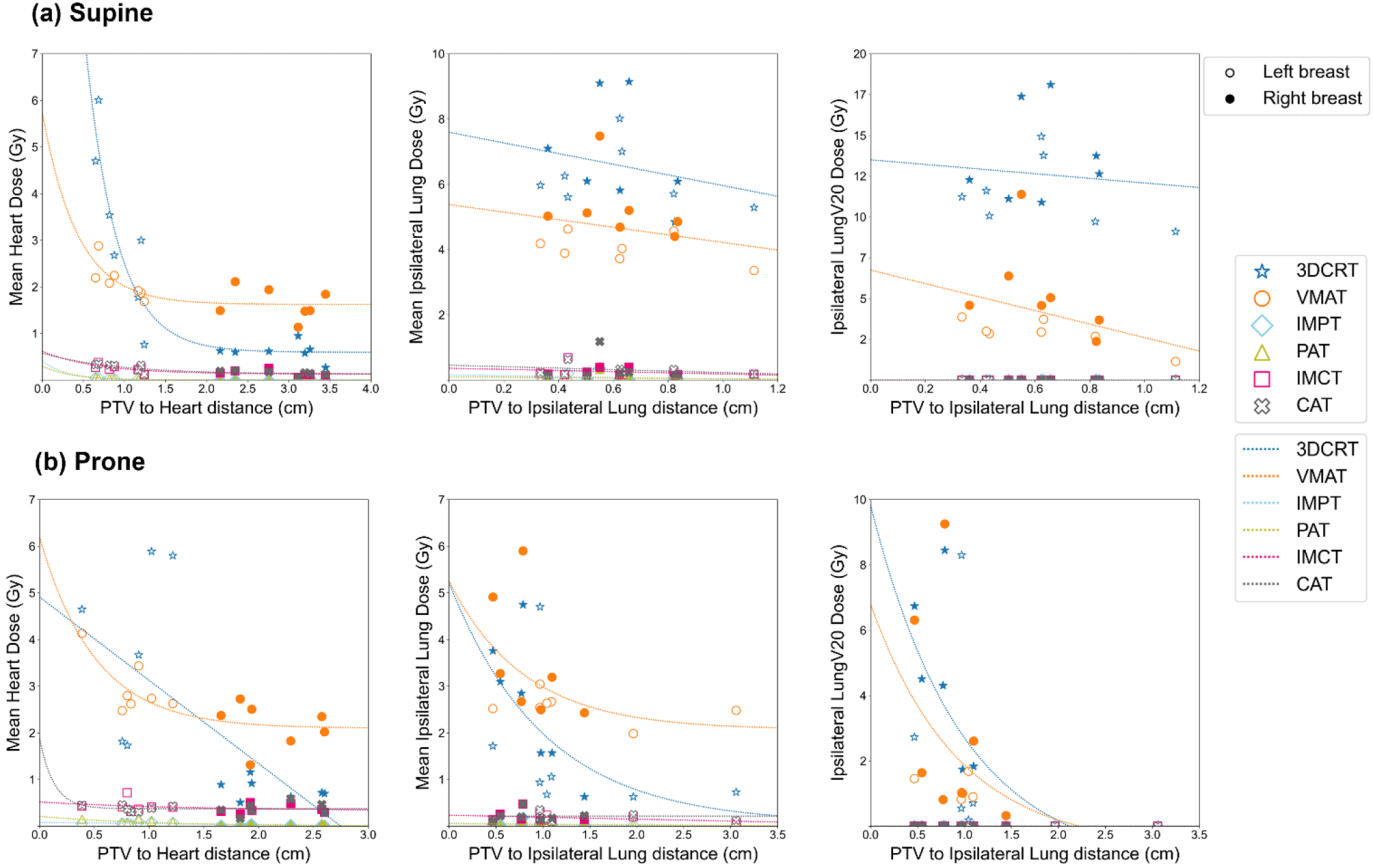
Relationship between PTV-OAR distance and normal tissue dose in supine (**a**) and prone positions (**b**). The left- and right-sided breasts are shown with empty and filled symbols, respectively.

The results of existing studies on the prone position are heterogeneous, primarily focusing on patients with larger breast volumes^40,58–62^. Improvements in cardiac dosimetry in the prone position are closely related to breast volume and were particularly effective in reducing cardiac dose in patients with breast volumes exceeding 750 or 1000 cm^3^^63,64^. The results of our study in XRT were consistent with previous research. The mean breast volume in the patient's prone position was 571.43 ± 294.78 cm^3^, and no significant improvement in cardiac dose was observed for the prone position. However, the prone position in XRT has been confirmed to reduce lung dose (as depicted in Fig. 3). The prone position can significantly reduce radiation exposure to the ipsilateral lung^58,60,61,64,65^, and may alleviate acute and late pulmonary toxicity^64,66^. A novelty of this study was the measurement of the shortest distances between the 3D contours of the PTV and OARs for highly conformal RT-suitable analyses such as VMAT. Consequently, we observed that the heart and ipsilateral lungs were positioned farther from the PTV in the supine and prone positions, respectively (as illustrated in Fig. 4). These findings resulted in dosimetric benefits attributed to the chosen treatment position in XRT (as shown in Fig. 6). Our study found that PBT consistently reduced doses to OARs regardless of treatment position. However, the prone position presents challenges such as decreased setup accuracy and larger inter-fractional motion compared to the supine position^52,59,67^. Considering the complexities and learning curves associated with prone radiotherapy, the supine position may be a more practical choice for PBT. Dosimetric improvements due to treatment position can vary depending on the treatment technique. While treatment position can influence dosimetric outcomes, patient characteristics such as breast size can also impact the consistency of patient positioning (setup reproducibility). This highlights the importance of tailoring treatment position selection to individual patients and the specific treatment technique being used.

Investigating secondary cancer risk in breast cancer patients is necessary in terms of improving long-term quality of life. In this study, VMAT demonstrated the highest EAR estimate, and PBT showed a significantly lower EAR compared to XRT (Supplementary Table S3). Furthermore, we compared proton therapy and CIRT in the context of whole-breast irradiation, with proton therapy yielding a smaller risk estimate compared to CIRT. This study is the first to analyze secondary cancer risk in CIRT for whole-breast irradiation, although the contribution of neutrons to the total dose was not included. In addition, the impact of the variable RBE on secondary cancer risk was not considered in the current model. Consequently, generalizing the results of this study may be challenging due to inherent biological uncertainties, model limitations in accounting for RBE in tumor initiation, and the presence of significant inter-patient variation^68,69^. Nevertheless, these findings are expected to provide valuable insights for clinicians and patients in their decision-making processes, particularly in the context of reducing the secondary cancer risk when considering PBT.

This study has several limitations. First, the study involved a relatively small number of patients from a single institution, which restricts the generalizability of the findings. In addition, our study did not investigate Intensity-modulated radiation therapy and various 3DCRT techniques. Further studies with a larger patient cohort are required to validate and expand upon the results. Second, although many factors affect radiotherapy's therapeutic effect, the experimental design of our study is limited. Recently, the application of the prone position and advanced treatment techniques has been increasing in regional nodal irradiation to protect normal tissues^70,71^. However, our study did not investigate regional nodal irradiation or simultaneously integrated boost techniques. Furthermore, various hypofractionated approaches were also not considered. In addition to considering treatment techniques and patient position, cost-efficient aspects such as overall treatment time may also be essential for clinical decisions. Third, the robustness of treatment plans and the potential impact of patient respiratory motion on beam delivery were not assessed in this study. Despite the documented dosimetric benefits of PBT, concerns about plan robustness in the face of treatment uncertainties could pose challenges to its clinical adoption. PBT may be more susceptible to patient movements, including respiratory motion. Further investigations are therefore necessary to understand the robustness of treatment techniques and positions in the context of anatomical variations, such as breathing motion or breast swelling. Fourth, this study did not consider respiration-guided techniques, including deep inspiration breath hold (DIBH), which have been shown to effectively reduce heart doses^58,59,61^. DIBH suggests a measurable improvement in the dosimetric outcomes of conventional approaches. However, it is important to consider that DIBH procedures generally require more treatment time than free-breathing radiotherapy^72^. Our study demonstrated that PBT achieved a mean heart dose of less than 0.5 Gy, indicating that PBT in free breathing could be a valuable technique for cardiac sparing in appropriately selected patients. Lastly, differences in dose calculation algorithms between photon and proton plans may have introduced bias into our results. In addition, potential variations in RBE could impact the robustness of our findings. Therefore, cautious interpretation of these results is warranted.

## Conclusions

To extend the life expectancy of breast cancer patients, radiation oncologists must prioritize the mitigation of radiation toxicity risks. Thoughtful consideration of radiation techniques and treatment positions can lead to reduced OAR doses and an enhancement in the management of treatment-related toxicity. The choice of the ideal radiotherapy technique for breast cancer should be personalized, considering both patient-specific characteristics and cost-effectiveness. This study provides crucial dosimetric insights that can significantly inform the decision-making process concerning treatment techniques and positions.

### Supplementary Information

Supplementary Tables.

## Data Availability

The datasets used and/or analyzed during the current study are available from the corresponding author upon reasonable request.
