# Supplementary material for: Dosimetric analysis of six whole-breast irradiation techniques in supine and prone positions
Source: Sci Rep. 2024 Jun 21;14:14347. doi: 10.1038/s41598-024-65461-y (PMC11192744; doi:10.1038/s41598-024-65461-y)
Supplement: Supplementary file 1 — Supplementary Tables. [file 41598_2024_65461_MOESM1_ESM.docx]

**Supplementary Table S1. Dose statistics for target volume and organ at risk**

|  |  | **RT Technique** | | | | | | **Statistical significance** | | | | | | | | | | | | | | |
| --- | --- | --- | --- | --- | --- | --- | --- | --- | --- | --- | --- | --- | --- | --- | --- | --- | --- | --- | --- | --- | --- | --- |
| **Parameter** | **Position** | **3DCRT** | **VMAT** | **IMPT** | **PAT** | **IMCT** | **CAT** | a | b | c | d | e | f | g | h | i | j | k | l | m | n | o |
| PTV PCI | Supine | **0.51 ± 0.08** | **0.77 ± 0.06** | **0.65 ± 0.07** | **0.61 ± 0.08** | 0.72 ± 0.05 | 0.66 ± 0.07 | x | x | x | x | x | x | x | x | x | x | x |  | x |  | x |
|  | Prone | **0.62 ± 0.10** | **0.82 ± 0.06** | **0.71 ± 0.07** | **0.67 ± 0.07** | 0.74 ± 0.04 | 0.68 ± 0.06 | x | x |  | x |  | x | x | x | x | x |  |  | x |  | x |
|  | *P* (supine vs. prone) | <.001 | <.001 | <.001 | <.001 | 0.057 | 0.179 |  |  |  |  |  |  |  |  |  |  |  |  |  |  |  |
|  |  |  |  |  |  |  |  |  |  |  |  |  |  |  |  |  |  |  |  |  |  |  |
| PTV HI | Supine | 6.83 ± 1.32 | 5.48 ± 0.99 | 5.44 ± 1.76 | 4.38 ± 1.80 | 6.52 ± 2.04 | **6.74 ± 1.58** |  |  | x |  |  |  |  |  |  |  |  |  |  | x |  |
|  | Prone | 6.93 ± 1.09 | 5.83 ± 0.80 | 5.63 ± 2.05 | 4.44 ± 1.45 | 5.71 ± 1.80 | **5.11 ± 1.01** | x |  | x |  | x |  | x |  |  | x |  |  |  |  |  |
|  | *P* (supine vs. prone) | 0.785 | 0.258 | 0.624 | 0.782 | 0.286 | 0.003 |  |  |  |  |  |  |  |  |  |  |  |  |  |  |  |
|  |  |  |  |  |  |  |  |  |  |  |  |  |  |  |  |  |  |  |  |  |  |  |
| Mean heart dose  (Gy) | Supine | 1.91 ± 1.80 | **1.88 ± 0.43** | **0.02 ± 0.02** | **0.01 ± 0.02** | **0.20 ± 0.09** | **0.22 ± 0.09** |  | x | x | x | x | x | x | x | x |  | x | x | x | x |  |
|  | Prone | 2.27 ± 1.95 | **2.57 ± 0.67** | **0.04 ± 0.03** | **0.06 ± 0.05** | **0.40 ± 0.11** | **0.38 ± 0.10** |  | x | x | x | x | x | x | x | x |  | x | x | x | x |  |
|  | *P* (supine vs. prone) | 0.33 | 0.001 | <.001 | <.001 | <.001 | <.001 |  |  |  |  |  |  |  |  |  |  |  |  |  |  |  |
|  |  |  |  |  |  |  |  |  |  |  |  |  |  |  |  |  |  |  |  |  |  |  |
| LAD max. dose  (Gy) | Supine | 21.52 ± 24.28 | 7.75 ± 6.80 | 4.36 ± 6.14 | 5.15 ± 7.51 | 2.48 ± 3.37 | 3.61 ± 5.48 | L | L | L | L | L |  |  | x |  |  |  |  |  |  |  |
|  | Prone | 23.41 ± 23.25 | 9.46 ± 8.02 | 5.04 ± 6.30 | 4.75 ± 5.36 | 2.66 ± 3.09 | 4.03 ± 4.41 | L | x | x | x | x | x | x | x | x |  |  |  |  |  |  |
|  | *P* (supine vs. prone) | 0.58 | 0.249 | 0.535 | 0.709 | 0.859 | 0.685 |  |  |  |  |  |  |  |  |  |  |  |  |  |  |  |
|  |  |  |  |  |  |  |  |  |  |  |  |  |  |  |  |  |  |  |  |  |  |  |
| Mean ipsilateral  lung dose (Gy) | Supine | **6.58 ± 1.34** | **4.66 ± 0.98** | **0.08 ± 0.10** | 0.06 ± 0.08 | 0.25 ± 0.15 | **0.32 ± 0.28** | x | x | x | x | x | x | x | x | x |  | x | x | x | x |  |
|  | Prone | **2.05 ± 1.50** | **3.05 ± 1.07** | **0.04 ± 0.04** | 0.04 ± 0.04 | 0.19 ± 0.10 | **0.20 ± 0.10** | x | x | x | x | x | x | x | x | x |  | x | x | x | x |  |
|  | *P* (supine vs. prone) | <.001 | <.001 | 0.025 | 0.058 | 0.078 | 0.048 |  |  |  |  |  |  |  |  |  |  |  |  |  |  |  |
|  |  |  |  |  |  |  |  |  |  |  |  |  |  |  |  |  |  |  |  |  |  |  |
| Ipsilateral Lung  V20 (%) | Supine | **12.62 ± 2.72** | **4.16 ± 2.45** | 0.00 ± 0.00 | 0.00 ± 0.00 | 0.00 ± 0.00 | 0.00 ± 0.00 | x | x | x | x | x | x | x | x | x |  |  |  |  |  |  |
|  | Prone | **2.86 ± 3.1** | **1.99 ± 2.61** | 0.00 ± 0.00 | 0.00 ± 0.00 | 0.00 ± 0.00 | 0.00 ± 0.00 |  |  |  |  |  |  |  |  |  |  |  |  |  |  |  |
|  | *P* (supine vs. prone) | <.001 | <.001 | 0.239 |  | 0.336 |  |  |  |  |  |  |  |  |  |  |  |  |  |  |  |  |
|  |  |  |  |  |  |  |  |  |  |  |  |  |  |  |  |  |  |  |  |  |  |  |
| Mean Contralateral  Breast Dose (Gy) | Supine | **0.57 ± 0.40** | **2.22 ± 0.94** | 0.03 ± 0.03 | **0.04 ± 0.02** | 0.11 ± 0.05 | **0.10 ± 0.05** | x | x | x | x | x | x | x | x | x |  | x | x | x | x |  |
|  | Prone | **0.97 ± 0.53** | **1.44 ± 0.59** | 0.05 ± 0.05 | **0.06 ± 0.03** | 0.15 ± 0.11 | **0.21 ± 0.09** | x | x | x | x | x | x | x | x | x |  | x | x | x | x |  |
|  | *P* (supine vs. prone) | 0.007 | 0.048 | 0.147 | 0.023 | 0.201 | 0.002 |  |  |  |  |  |  |  |  |  |  |  |  |  |  |  |
|  |  |  |  |  |  |  |  |  |  |  |  |  |  |  |  |  |  |  |  |  |  |  |
| Skin D2 (Gy) | Supine | 50.57 ± 0.79 | **49.09 ± 0.32** | 51.47 ± 0.73 | **51.63 ± 0.47** | 51.99 ± 1.00 | 53.15 ± 0.94 | x |  | x | x | x | x | x | x | x |  |  | x |  | x |  |
|  | Prone | 50.49 ± 0.67 | **49.51 ± 0.62** | 51.40 ± 0.64 | **51.37 ± 0.43** | 51.9 ± 0.46 | 52.74 ± 0.51 | x | x | x | x | x | x | x | x | x |  |  | x | x | x | x |
|  | *P* (supine vs. prone) | 0.575 | 0.021 | 0.629 | 0.009 | 0.743 | 0.087 |  |  |  |  |  |  |  |  |  |  |  |  |  |  |  |

3DCRT: Three-dimensional Conformal Radiotherapy; VMAT: Volumetric-modulated Arc Therapy; IMPT: Intensity-modulated Proton Therapy; PAT: Proton Arc Therapy; IMCT: Intensity-modulated Carbon-ion Therapy; CAT: Carbon-ion Arc Therapy

PCI: Paddick conformity index; HI: Homogeneity index

Values for RT techniques are expressed as the mean with standard deviation.

x indicates statistical significance determined by paired *t* test for RT techniques comparison (a = 3DCRT versus VMAT; b = 3DCRT versus IMPT; c = 3DCRT versus PAT; d = 3DCRT versus IMCT; e = 3DCRT versus CAT; f = VMAT versus IMPT; g = VMAT versus PAT; h = VMAT versus IMCT; I = VMAT versus CAT; j = IMPT versus PAT; k = IMPT versus IMCT; l = IMPT versus CAT; m = PAT versus IMCT; n = PAT versus CAT; o = IMCT versus CAT). L indicates statistically significant difference only in left breast cancer patients. Values in bold indicate statistically significant association in patient position (supine versus prone)

**Supplementary Table S2. Geometric differences between supine and prone positions**

|  | Supine | Prone | *P*-value |
| --- | --- | --- | --- |
| PTV volume (cm^3^) | 486.56 ± 237.02 | 571.43 ± 294.78 | 0.004 |
| HSD (mm) | 19.23 ± 10.79 | 14.81 ± 7.29 | 0.015 |
| ILSD (mm) | 6.21 ± 2.17 | 11.19 ± 6.82 | 0.003 |

PTV: planning target volume; HSD: heart separation distance; ILSD: ipsilateral separation distance

**Supplementary Table S3. Estimated absolute risks**

1. **Contralateral breast**

| **Parameter** | **Position** | **RT Technique** | | | | | | **Statistical significance** | | | | | | | | | | | | | | | |
| --- | --- | --- | --- | --- | --- | --- | --- | --- | --- | --- | --- | --- | --- | --- | --- | --- | --- | --- | --- | --- | --- | --- | --- |
|  |  | **3DCRT** | **VMAT** | **IMPT** | **PAT** | **IMCT** | **CAT** | a | b | c | d | e | f | g | h | i | j | k | l | m | n | o |  |
| OED | Supine | **0.52 ± 0.21** | **1.92 ± 0.25** | 0.03 ± 0.03 | **0.04 ± 0.02** | 0.11 ± 0.05 | **0.10 ± 0.05** | x | x | x | x | x | x | x | x | x |  | x | x | x | x |  |  |
|  | Prone | **0.77 ± 0.34** | **1.13 ± 0.11** | 0.05 ± 0.04 | **0.06 ± 0.03** | 0.15 ± 0.11 | **0.20 ± 0.09** | x | x | x | x | x | x | x | x | x |  | x | x |  | x |  |  |
|  | *P* (supine vs. prone) | 0.005 | <.001 | 0.128 | 0.023 | 0.209 | 0.002 |  |  |  |  |  |  |  |  |  |  |  |  |  |  |  |  |
|  |  |  |  |  |  |  |  |  |  |  |  |  |  |  |  |  |  |  |  |  |  |  |  |
| EAR | Supine | **4.28 ± 1.75** | **15.76 ± 2.09** | 0.26 ± 0.22 | **0.33 ± 0.20** | 0.89 ± 0.41 | **0.84 ± 0.41** | x | x | x | x | x | x | x | x | x |  | x | x | x | x |  |  |
|  | Prone | **6.32 ±2.76** | **9.23 ± 0.94** | 0.45 ± 0.36 | **0.51 ± 0.27** | 1.25 ± 0.92 | **1.65 ± 0.74** | x | x | x | x | x | x | x | x | x |  | x | x |  | x |  |  |
|  | *P* (supine vs. prone) | 0.005 | <.001 | 0.128 | 0.023 | 0.209 | 0.002 |  |  |  |  |  |  |  |  |  |  |  |  |  |  |  |  |

OED: Organ Effective Dose; EAR: excess absolute risk; 3DCRT: Three-dimensional Conformal Radiotherapy; VMAT: Volumetric-modulated Arc Therapy; IMPT: Intensity-modulated Proton Therapy; PAT: Proton Arc Therapy; IMCT: Intensity-modulated Carbon-ion Therapy; CAT: Carbon-ion Arc Therapy.

x indicates statistical significance determined by paired t-test for RT techniques comparison (a = 3DCRT versus VMAT; b = 3DCRT versus IMPT; c = 3DCRT versus PAT; d = 3DCRT versus IMCT; e = 3DCRT versus CAT; f = VMAT versus IMPT; g = VMAT versus PAT; h = VMAT versus IMCT; I = VMAT versus CAT; j = IMPT versus PAT; k = IMPT versus IMCT; l = IMPT versus CAT; m = PAT versus IMCT; n = PAT versus CAT; o = IMCT versus CAT).

1. **Ipsilateral lung**

| **Parameter** | **Position** | **RT Technique** | | | | | | **Statistical significance** | | | | | | | | | | | | | | | |
| --- | --- | --- | --- | --- | --- | --- | --- | --- | --- | --- | --- | --- | --- | --- | --- | --- | --- | --- | --- | --- | --- | --- | --- |
|  |  | **3DCRT** | **VMAT** | **IMPT** | **PAT** | **IMCT** | **CAT** | a | b | c | d | e | f | g | h | i | j | k | l | m | n | o |  |
| OED | Supine | **3.59 ± 0.52** | **3.35 ± 0.48** | **0.07 ± 0.09** | **0.06 ± 0.07** | 0.25 ± 0.14 | **0.3 ± 0.26** |  | x | x | x | x | x | x | x | x |  | x | x | x | x |  |  |
|  | Prone | **1.30 ± 0.72** | **2.34 ± 0.57** | **0.03 ± 0.04** | **0.04 ± 0.03** | 0.18 ± 0.10 | **0.2 ± 0.10** | x | x | x | x | x | x | x | x | x |  | x | x | x | x |  |  |
|  | *P* (supine vs. prone) | >0.001 | >0.001 | 0.022 | 0.046 | 0.082 | 0.044 |  |  |  |  |  |  |  |  |  |  |  |  |  |  |  |  |
|  |  |  |  |  |  |  |  |  |  |  |  |  |  |  |  |  |  |  |  |  |  |  |  |
| EAR | Supine | **28.71 ± 4.13** | **26.83 ± 3.88** | **0.57 ± 0.69** | **0.47 ± 0.56** | 1.98 ± 1.1 | **2.44 ± 2.07** |  | x | x | x | x | x | x | x | x |  | x | x | x | x |  |  |
|  | Prone | **10.39 ± 5.79** | **18.73 ± 4.58** | **0.28 ± 0.31** | **0.3 ± 0.27** | 1.48 ± 0.82 | **1.58 ± 0.79** | x | x | x | x | x | x | x | x | x |  | x | x | x | x |  |  |
|  | *P* (supine vs. prone) | >0.001 | >0.001 | 0.022 | 0.046 | 0.082 | 0.044 |  |  |  |  |  |  |  |  |  |  |  |  |  |  |  |  |

OED: Organ Effective Dose; EAR: excess absolute risk; 3DCRT: Three-dimensional Conformal Radiotherapy; VMAT: Volumetric-modulated Arc Therapy; IMPT: Intensity-modulated Proton Therapy; PAT: Proton Arc Therapy; IMCT: Intensity-modulated Carbon-ion Therapy; CAT: Carbon-ion Arc Therapy.

x indicates statistical significance determined by paired t-test for RT techniques comparison (a = 3DCRT versus VMAT; b = 3DCRT versus IMPT; c = 3DCRT versus PAT; d = 3DCRT versus IMCT; e = 3DCRT versus CAT; f = VMAT versus IMPT; g = VMAT versus PAT; h = VMAT versus IMCT; I = VMAT versus CAT; j = IMPT versus PAT; k = IMPT versus IMCT; l = IMPT versus CAT; m = PAT versus IMCT; n = PAT versus CAT; o = IMCT versus CAT).

1. **Contralateral lung**

| **Parameter** | **Position** | **RT Technique** | | | | | | **Statistical significance** | | | | | | | | | | | | | | | |
| --- | --- | --- | --- | --- | --- | --- | --- | --- | --- | --- | --- | --- | --- | --- | --- | --- | --- | --- | --- | --- | --- | --- | --- |
|  |  | **3DCRT** | **VMAT** | **IMPT** | **PAT** | **IMCT** | **CAT** | a | b | c | d | e | f | g | h | i | j | k | l | m | n | o |  |
| OED | Supine | 0.20 ± 0.06 | 1.85 ± 0.72 | 0.00 ± 0.00 | 0.02 ± 0.05 | **0.02 ± 0.01** | **0.06 ± 0.03** | x | x | x | x | x | x | x | x | x |  | x | x |  |  | x |  |
|  | Prone | 0.18 ± 0.08 | 2.12 ± 0.68 | 0.00 ± 0.00 | 0.01 ± 0.01 | **0.08 ± 0.04** | **0.1 ± 0.04** | x | x | x | x | x | x | x | x | x |  | x | x | x | x |  |  |
|  | *P* (supine vs. prone) | 0.156 | 0.313 | 0.834 | 0.462 | <0.001 | <0.001 |  |  |  |  |  |  |  |  |  |  |  |  |  |  |  |  |
|  |  |  |  |  |  |  |  |  |  |  |  |  |  |  |  |  |  |  |  |  |  |  |  |
| EAR | Supine | 1.62 ± 0.49 | 14.83 ± 5.78 | 0.01 ± 0.01 | 0.16 ± 0.41 | **0.15 ± 0.11** | **0.50 ± 0.21** | x | x | x | x | x | x | x | x | x |  | x | x |  |  | x |  |
|  | Prone | 1.42 ± 0.62 | 16.97 ± 5.44 | 0.00 ± 0.01 | 0.07 ± 0.09 | **0.63 ± 0.31** | **0.79 ± 0.31** | x | x | x | x | x | x | x | x | x |  | x | x | x | x |  |  |
|  | *P* (supine vs. prone) | 0.156 | 0.313 | 0.834 | 0.462 | <0.001 | <0.001 |  |  |  |  |  |  |  |  |  |  |  |  |  |  |  |  |

OED: Organ Effective Dose; EAR: excess absolute risk; 3DCRT: Three-dimensional Conformal Radiotherapy; VMAT: Volumetric-modulated Arc Therapy; IMPT: Intensity-modulated Proton Therapy; PAT: Proton Arc Therapy; IMCT: Intensity-modulated Carbon-ion Therapy; CAT: Carbon-ion Arc Therapy.

x indicates statistical significance determined by paired t-test for RT techniques comparison (a = 3DCRT versus VMAT; b = 3DCRT versus IMPT; c = 3DCRT versus PAT; d = 3DCRT versus IMCT; e = 3DCRT versus CAT; f = VMAT versus IMPT; g = VMAT versus PAT; h = VMAT versus IMCT; I = VMAT versus CAT; j = IMPT versus PAT; k = IMPT versus IMCT; l = IMPT versus CAT; m = PAT versus IMCT; n = PAT versus CAT; o = IMCT versus CAT).
